# Association of circulating immuno-oncology biomarkers with breast cancer risk: insights from two prospective cohorts

**DOI:** 10.1038/s41698-025-01019-z

**Published:** 2025-07-15

**Authors:** Jing Zheng, Chen Suo, Yan Wu, Baokun Cao, Ziyu Yuan, Yanfeng Jiang, Zhenqiu Liu, Weimin Ye, Haomin Yang, Xingdong Chen

**Affiliations:** 1 Department of Epidemiology and Health Statistics, School of Public Health, Fujian Medical University, Fuzhou, China; 2 Department of Epidemiology, School of Public Health, and the Key Laboratory of Public Health Safety of Ministry of Education, Fudan University, Shanghai, China; 3 Fudan University Taizhou Institute of Health Sciences, Taizhou, Jiangsu, China; 4 Department of Health Inspection and Quarantine, School of Public Health, Fujian Medical University, Fuzhou, China; 5 State Key Laboratory of Genetic Engineering, Human Phenome Institute, and School of Life Sciences, Fudan University, Shanghai, China; 6 Department of Medical Epidemiology and Biostatistics, Karolinska Institutet, Stockholm, Sweden

**Keywords:** Predictive markers, Breast cancer, Cancer epidemiology, Tumour biomarkers

## Abstract

Immuno-oncology biomarkers are promising tools for cancer risk assessment and early detection. To identify and validate their associations with breast cancer, nested case-control studies within the Taizhou Longitudinal Study (TZL) cohort and the UK biobank Pharma Proteomics Project (UKB-PPP) were conducted, comprising 195 and 881 incident breast cancer patients, together with their matched controls. Among the 92 plasma proteins tested by the Olink Immuno-oncology panel, 11 proteins were associated with breast cancer risk in the TZL cohort after multiple testing correction. Notably, hepatocyte growth factor (HGF) was validated in the UKB-PPP, particularly among postmenopausal women (OR = 1.13, 95% CI = 1.03–1.24). The association was stronger with Estrogen Receptor-negative breast cancer and confirmed by Mendelian Randomization analysis. Additionally, HGF mediated the effects of Healthy Lifestyle Index (27.17%) and BMI (19.79%) on breast cancer risk. Therefore, HGF could be an intervention target by either medicines or lifestyle changes to improve the prevention and treatment of breast cancer.

## Introduction

Breast cancer remains the most prevalent cancer among women, accounting for 11.6% of all cancers, with an annual incidence of 2.3 million^1^. The global burden of breast cancer has seen a substantial rise from 1990 to 2021, particularly among younger populations, which presents a considerable public health challenge^2^. Early detection is crucial for improving survival rates, with mammographic imaging being the primary method for breast cancer screening^3^. However, mammography has challenges, including high costs, reliance on radiologists’ skill, and risks of false positives and negatives that can lead to unnecessary procedures and anxiety for patients^4,5^. Additionally, common tumor markers used in clinical practice, such as carbohydrate antigen 15-3 (CA 15-3) and carcinoembryonic antigen (CEA), have limited sensitivity and specificity^6,7^. These limitations highlight the need for discovering new biomarkers for breast cancer.

Circulating protein biomarkers are promising for cancer detection and risk assessment due to their simplicity, minimal invasiveness, and biological stability^8^. However, most of the proteomics studies on breast cancer used blood samples after the diagnosis of the disease, which might be affected by diagnosis procedures and treatment effects^9–11^. The only prospective study reported borderline significant association of Caspase-8 (CASP8) with short-term breast cancer risk^12^. In addition, relatively few studies provide sufficient cross validations for the associations^13,14^.

Besides the proteins involved in the tumor growth and apoptosis pathways, which are potentially related to the short-term risk of breast cancer, immunity and inflammation are other significant risk factors in breast cancer development. Chronic inflammation not only impairs immune surveillance, but also modifies signaling pathways linked to carcinogenesis^15–17^. To improve predictive applications, it is crucial to conduct a comprehensive assessment of the immuno-oncology biomarkers for both the short-term and long-term risk of breast cancer.

This study aims to prospectively identify immuno-oncology proteins for breast cancer risk, and assess the short-term and long-term effects in Taizhou Longitudinal Study (TZL) cohort in China. We will further validate the findings in UK biobank Pharma Proteomics Project (UKB-PPP) to enhance the generalizability of our results for diverse populations. Additionally, we will evaluate the potential genetic causal effects of these proteins.

## Results

**Fig. 1:**
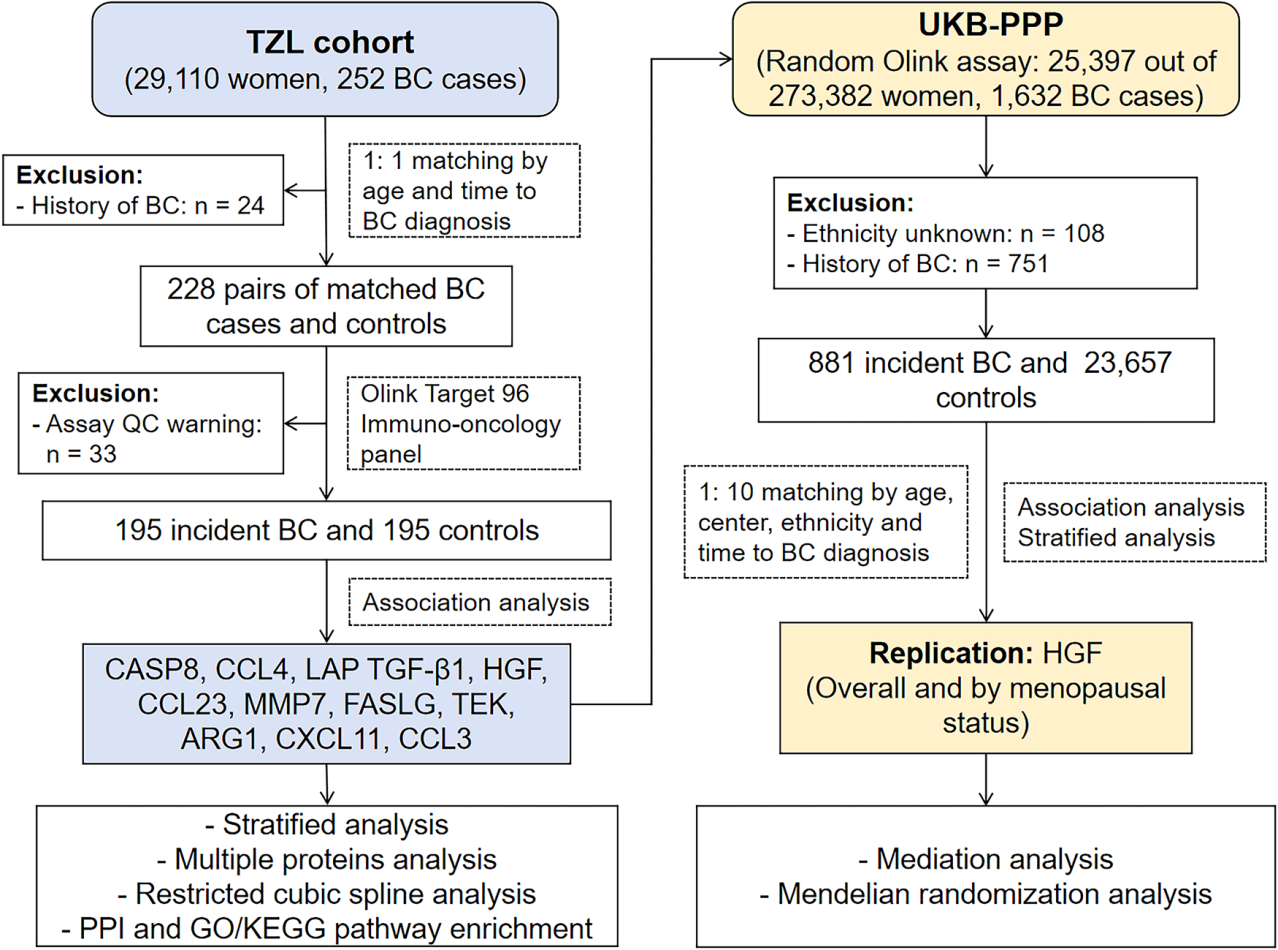
Flowchart of the study design.

The detailed workflow of this study is presented in Fig. 1. Following rigorous QC measures, 195 pairs of newly diagnosed breast cancer cases and matched controls were included in subsequent analyses in the TZL cohort. The mean age of participants was 53.99 years (SD 9.58). Among breast cancer cases, the median time from enrollment to diagnosis was 5.47 years (IQR 4.89), with a mean diagnosis age of 59.03 years (SD 9.92). Notably, the case group demonstrated significantly higher incomes and greater heights compared to matched controls. No statistically significant differences were observed between the two groups in terms of alcohol consumption, smoking status, BMI, waist circumference, menopausal status, number of births, and family history of breast cancer (Table 1).

**Table 1:** Baseline characteristics of participants in the TZL cohort (*N* = 390)

Characteristic	Cases, *N* (%)	Controls, *N* (%)	*P*	
Age at blood draw (years), mean (SD)	53.99 (9.58)	53.99 (9.58)	–	
Time to cancer diagnosis (years), Median (IQR)	5.47 (4.89)	–	–	
*Income (CNY/year)*				
<10,000	13 (6.67%)	44 (22.56%)	Ref	
10,000–20,000	16 (8.20%)	22 (11.28%)	0.110	
20,000–35,000	36 (18.46%)	35 (17.95%)	**0.004**	
≥35,000	130 (66.67%)	94 (48.21%)	**<0.001**	
*Alcohol consumption*				
Never	183 (93.85%)	184 (94.36%)	Ref	
Former	2 (1.02%)	2 (1.02%)	0.971	
Current	10 (5.13%)	9 (4.62%)	0.751	
*Smoking*				
Never	194 (99.49%)	193 (98.98%)	Ref	
Former	0 (0.00%)	1 (0.51%)	0.990	
Current	1 (0.51%)	1 (0.51%)	>0.999	
BMI (kg/m^2^), mean (SD)	25.18 (3.88)	24.56 (3.49)	0.090	
*BMI (kg/m*^2^*)*				
<24	78 (40.00%)	89 (45.64%)	Ref	
≥24	117 (60.00%)	106 (54.36%)	0.264	
*Waist (cm)*				
<85	113 (57.95%)	127 (65.13%)	Ref	
≥85	82 (42.05%)	68 (34.87%)	0.159	
Height, mean (SD)	156.88 (5.48)	155.29 (5.68)		**0.009**
Age at menarche (years), mean (SD)	16.00 (2.20)	16.94 (2.48)		**<0.001**
*Age at menarche (years)*				
<14	25 (12.82%)	18 (9.23%)		Ref
≥14	170 (87.18%)	177 (90.77%)		0.477
*Menopausal status*				
Premenopausal	63 (32.31%)	70 (35.90%)		Ref
Postmenopausal	132 (67.69%)	125 (64.10%)		0.244
*Number of births*				
1	99 (50.77%)	84 (43.08%)		Ref
≥ 2	96 (49.23%)	111 (56.92%)		0.155
*Family history of breast cancer*				
No	194 (99.49%)	193 (98.97%)		Ref
Yes	1 (0.51%)	2 (1.03%)		0.742

Data shown are mean (SD) or median (IQR) for continuous variables and percent for categorical variables, using multiply imputed data.Statistically significant differences are in bold.

### Association of proteins with breast cancer in the TZL Cohort

**Fig. 2:**
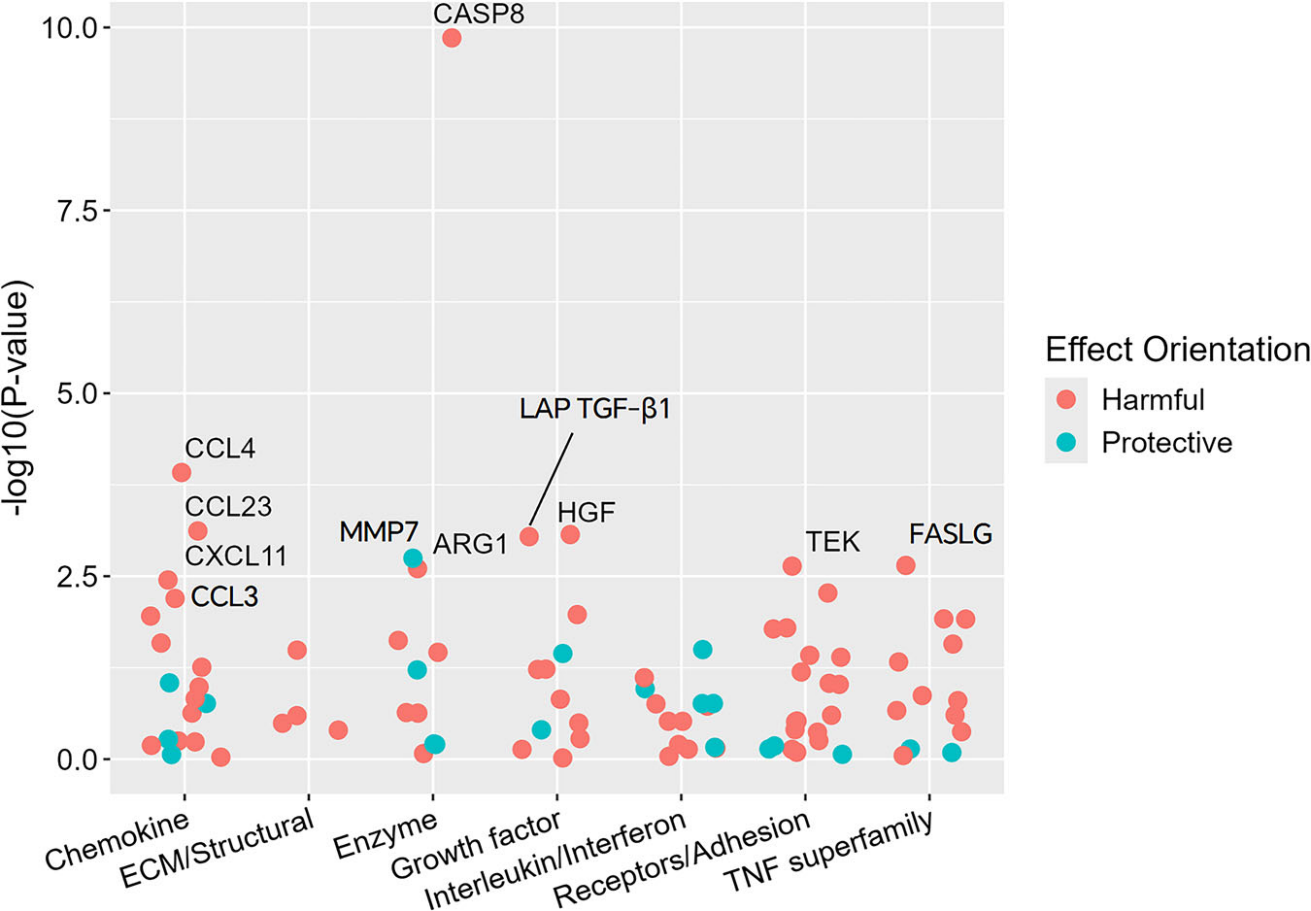
Association of 92 proteins in the targeted panel with incident breast cancer (*N* = 390). Adjusted for income, age at menarche, menopausal status, number of births, BMI and height. Eleven proteins were found to be significantly associated with incident breast cancer risk after accounting for multiple testing (FDR < 0.05).

**Fig. 3:**
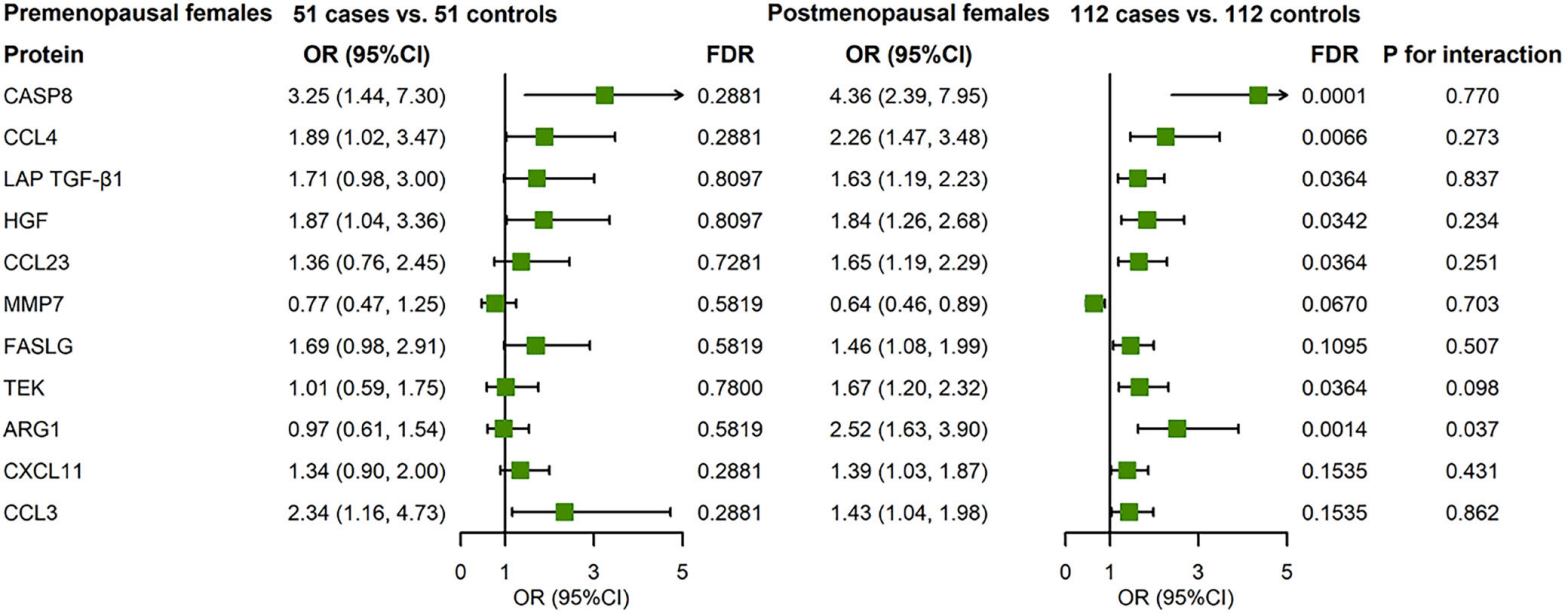
Associations between 11 proteins and incident breast cancer stratified by menopausal status in the TZL cohort. Adjusted for income, age at menarche, number of births, BMI and height.

After adjusting for potential confounders and using an FDR threshold of <0.05, 11 of the 92 immuno-oncology proteins were significantly associated with breast cancer. The adjusted odds ratios (ORs) for a 1 standard deviation (SD) increase in protein levels ranged from 1.38 (C–X–C motif chemokine 11, CXCL11: 95% CI = 1.11–1.72) to 3.71 (CASP8: 95% CI = 2.49–5.54) for the 10 proteins exhibiting positive associations, while Matrix metalloproteinase-7 (MMP7) displayed an inverse association (OR = 0.69, 95% CI = 0.55–0.87) (Figs. 2 and 3). Additional RCS showed clear trends for the associations, especially beyond the mean value (Fig. S1), and sensitivity analyses indicated little difference between the original and multiple imputation datasets (Table S1).

**Fig. 4:**
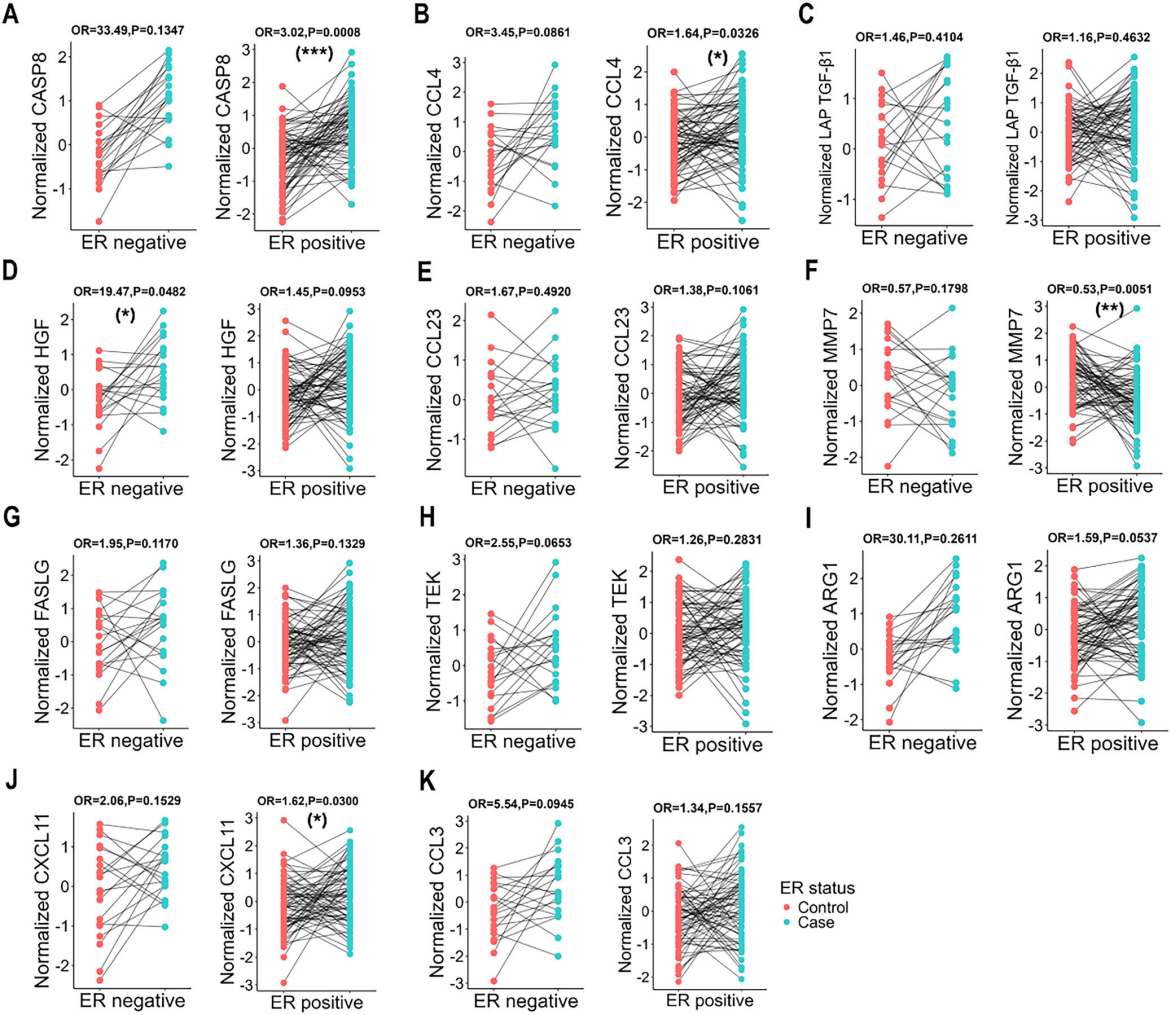
Associations between 11 proteins and incident breast cancer stratified by tumor ER status in the TZL cohort study (ER negative: 20 cases; ER positive: 69 cases). Adjusted for age at menarche, menopausal status, number of births, BMI and height. The proteins analyzed include: CASP8 (**A**), CCL4 (B), LAP TGF-β1 (**C**), HGF (**D**), CCL23 (**E**), MMP7 (**F**), FASLG (**G**), TEK (**H**), ARG1 (**I**), CXCL11 (**J**), and CCL3 (**K**). Statistical significance is denoted as follows: *P < 0.05, **P < 0.01, and ***P < 0.001.

Stratified analysis by menopausal status revealed significant associations for 7 proteins among postmenopausal women. Notably, FAS ligand (FASLG), CXCL11, and C–C motif chemokine 3 (CCL3) did not demonstrate significant differences. In contrast, no significant associations were observed among premenopausal women, likely due to the limited sample size of premenopausal breast cancer (Fig. 3). Subsequent stratified analysis included a total of 89 cases with complete tumor pathological characteristics. The association of hepatocyte growth factor (HGF) with breast cancer was stronger in Estrogen Receptor (ER)-negative patients, while CASP8, CXCL11, MMP7, and C–C motif chemokine 4 (CCL4) were associated with ER-positive breast cancer (Fig. 4). Additionally, the relationship between Arginase-1 (ARG1) and advanced-stage breast cancer was more pronounced than in the early-stage group (Fig. S2). Similarly, this protein exhibited a stronger association in cases with larger tumor sizes and those with lymph node metastasis compared to tumors of smaller sizes and those without lymph node involvement (Figs. S3 and 4).

**Fig. 5:**
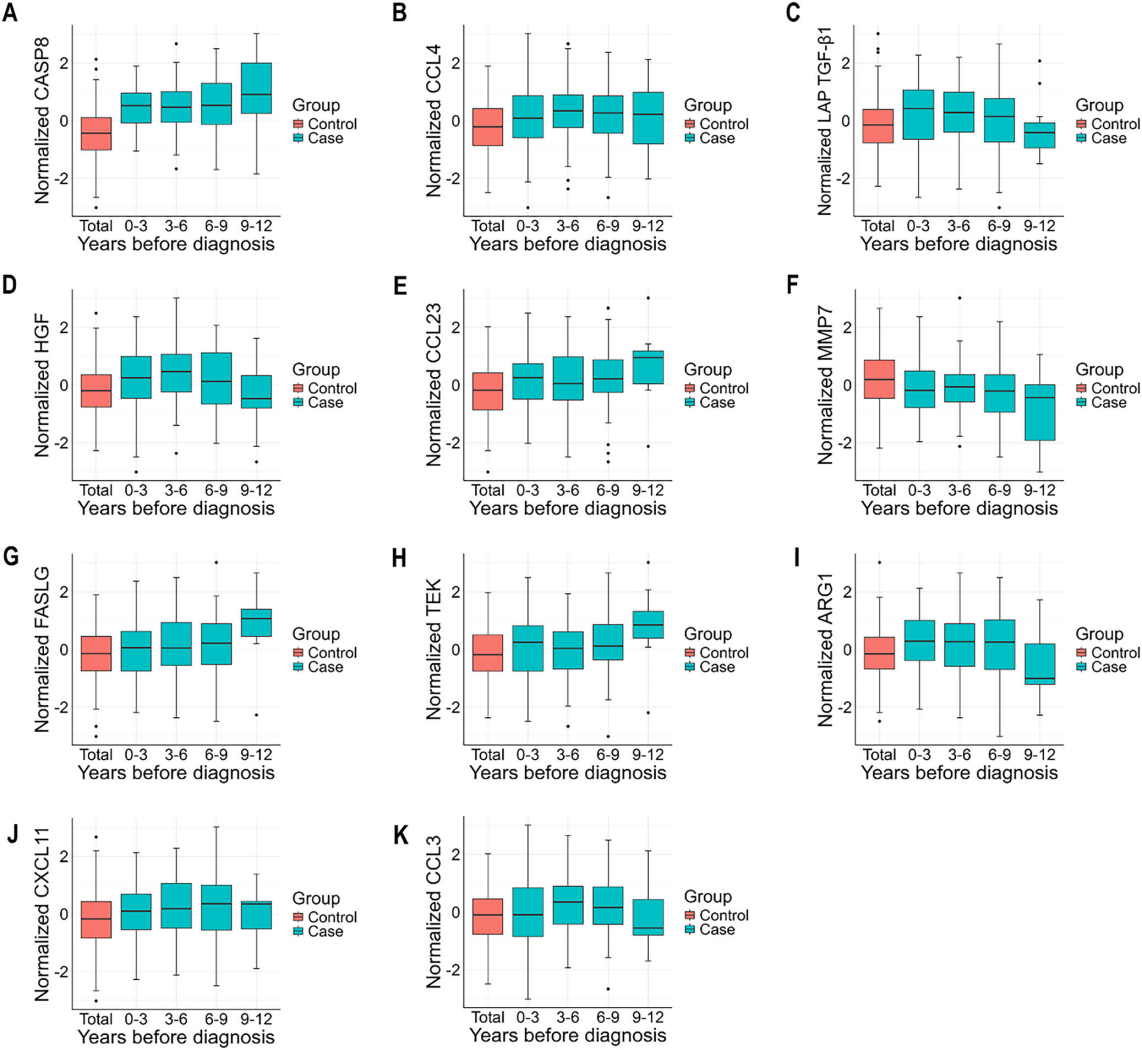
The box plots illustrate the distributions of the 11 identified proteins based on the years before breast cancer diagnosis within the TZL cohort, organized by three-year intervals. The proteins analyzed include: CASP8 (**A**), CCL4 (**B**), LAP TGF-β1 (**C**), HGF (**D**), CCL23 (**E**), MMP7 (**F**), FASLG (**G**), TEK (**H**), ARG1 (**I**), CXCL11 (**J**), and CCL3 (**K**).

Further stratification was conducted to investigate the impact of the time to breast cancer diagnosis. The analysis revealed no significant differences in the levels of proteins associated with breast cancer, regardless of whether the patients were diagnosed within 5 years or beyond 5 years of follow-up. Notably, elevated levels of CASP8, latency-associated peptide of transforming growth factor beta-1 (LAP TGF-β1), HGF, and ARG1 were observed among breast cancer patients diagnosed within 5 years of follow-up (Fig. S5). Box plots showing the distribution of protein levels according to the years prior to breast cancer diagnosis suggested similar results (Fig. 5), and further stratification by menopausal status revealed similar protein expression patterns in postmenopausal women (Figs. S6 and S7).

To further explore potential synergistic effects, we examined protein-protein interactions within their respective functional pathways. The results revealed significant interactions between LAP TGF-β1 and HGF (Table S2-3). Besides, the PPI network demonstrated the interaction strengths among 11 differentially expressed proteins, highlighting that TGF-β1 interacts with nine of these proteins, while HGF and FASLG each interact with eight. Furthermore, GO analysis indicated enrichment in immune and cellular responses, including cell migration, chemotaxis, and inflammatory responses. Similarly, KEGG terms highlighted the involvement of these proteins in host-virus interactions, cytokine signaling, and immune system processes (Fig. S8).

### Validation analysis in UKB-PPP

**Fig. 6:**
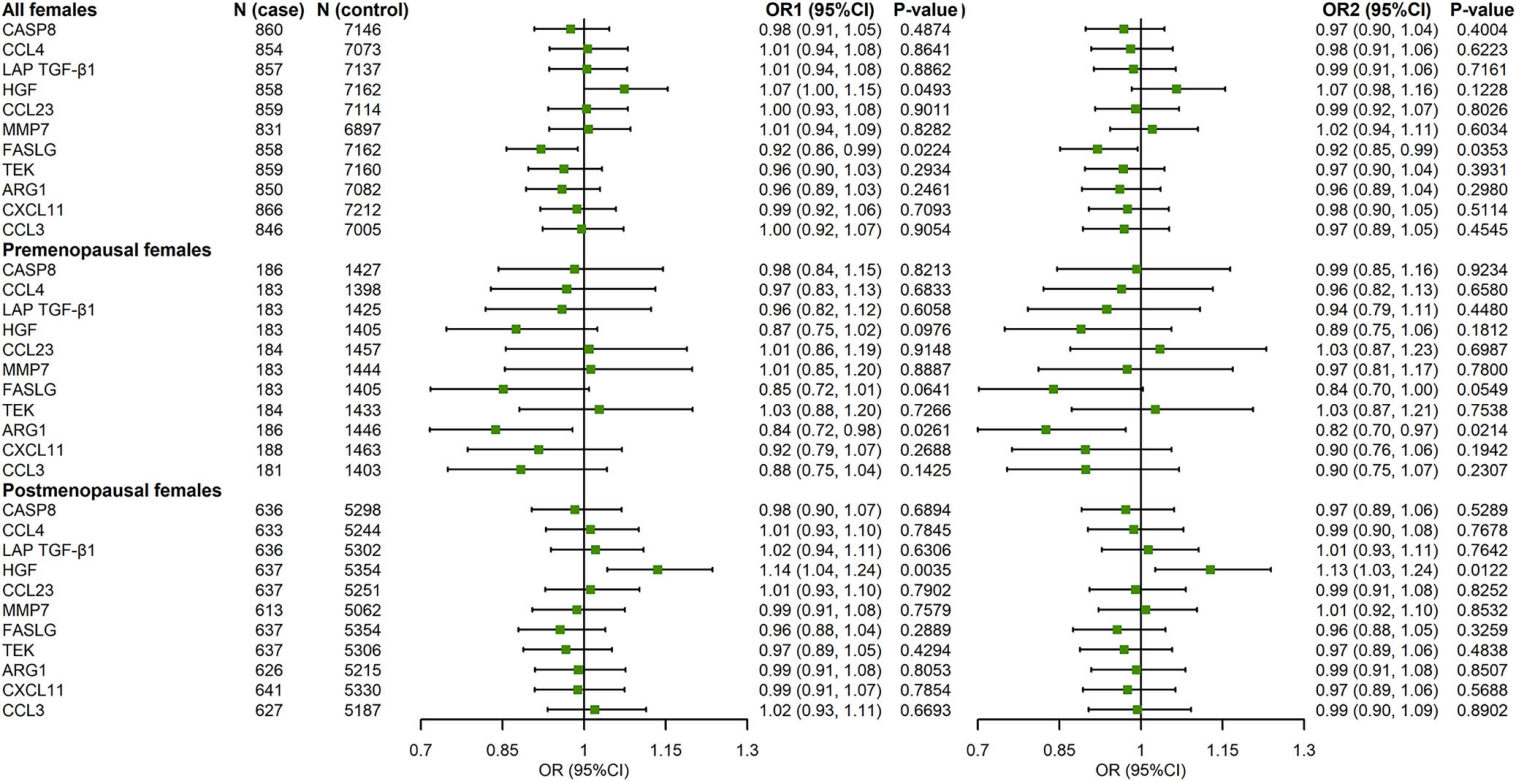
Associations of 11 proteins with incident breast cancer stratified by menopausal status in UKB-PPP. OR1 univariate analysis, OR2 multivariate analysis adjusted for TDI, alcohol consumption, smoking status, age at menarche, menopausal status (for all females), number of births, BMI, height, HRT history, family history of breast cancer, and fasting time at blood draw.

Among these proteins, only HGF was successfully validated. In the overall female population, univariate analysis indicated a significant association for HGF (1.07, 1.00–1.15); this association was borderline significant after adjusting for covariates (1.07, 0.98–1.16). Stratified analysis by menopausal status revealed significant associations for HGF among postmenopausal women (1.13, 1.03–1.24) after covariate adjustment (Fig. 6). Further stratification by time before breast cancer diagnosis demonstrated that among postmenopausal women diagnosed within five years, HGF remained significantly associated with breast cancer (1.19, 1.03–1.37) (Fig. S9).

**Fig. 7:**
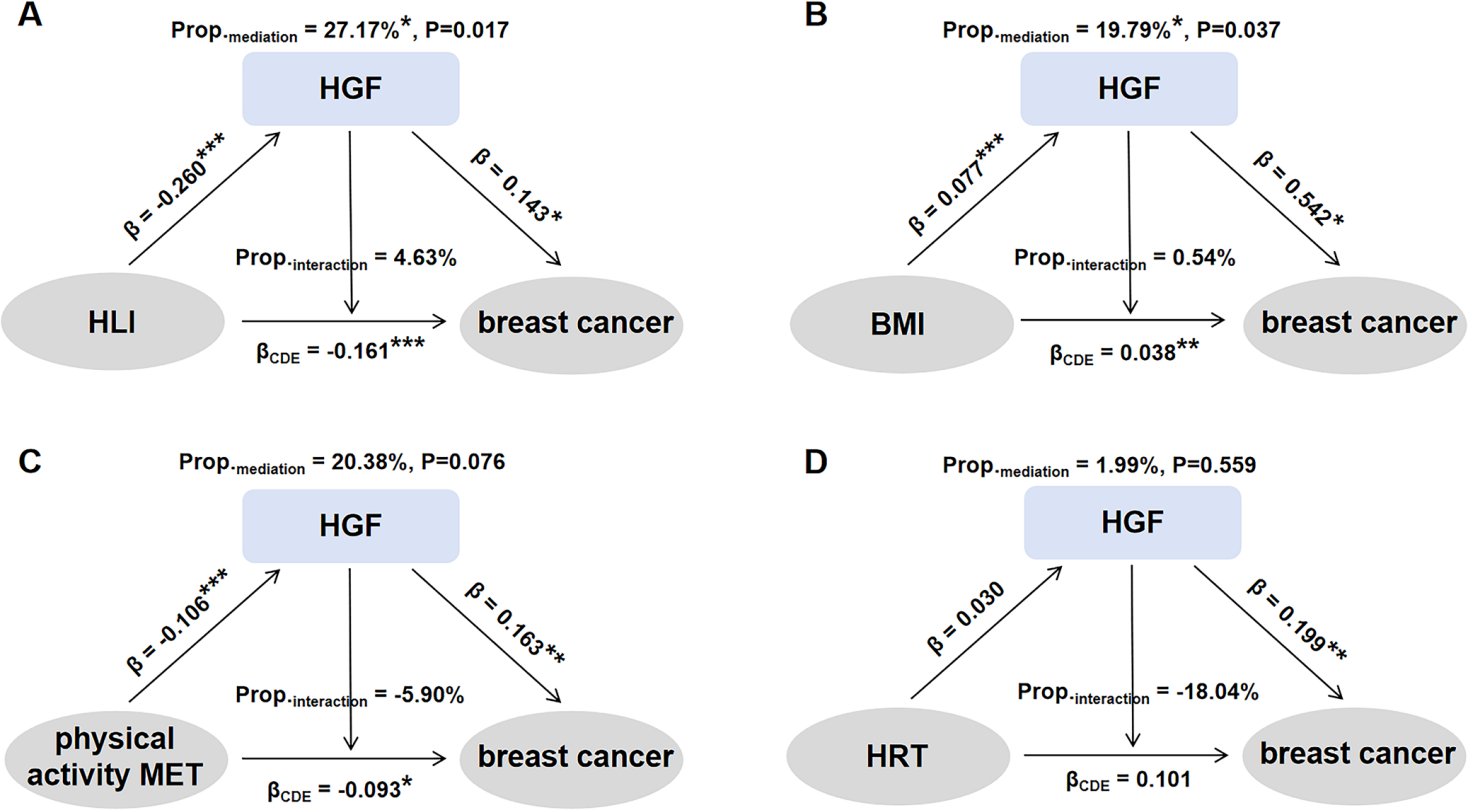
HGF is proposed to mediate the effect of the Healthy Lifestyle Index (HLI) and BMI on breast cancer risk among postmenopausal females in UKB-PPP. Four categories of exposures were considered: **A** HLI, **B** BMI, **C** physical activity measured in metabolic equivalent of task (MET), and **D** history of HRT. The analyses were adjusted for various factors, including TDI, age at menarche, menopausal status, number of births, height, HRT history, family history of breast cancer, and fasting time at blood draw. The HLI was not adjusted for its components (alcohol consumption, smoking status, and BMI). Statistical significance is denoted as follows: *P < 0.05, **P < 0.01, and ***P < 0.001.

Notably, the mediation analysis in UKB-PPP showed that HGF significantly mediated the effects of HLI (27.17%) and BMI (19.79%), as well as a marginally significant mediation effect concerning physical activity measured in MET (20.38%) on breast cancer risk. However, it did not mediate the associations of HRT with the risk of breast cancer (Fig. 7, Table S4).

### Mendelian randomization analysis

To investigate the causal relationship between HGF and breast cancer, an MR analysis was conducted using summary statistics of breast cancer from BCAC consortium and pQTLs from the UKB-PPP. A potential causal effect of HGF on breast cancer was observed using IVs with suggestive GWAS significance (OR = 1.05, 95% CI = 1.00–1.09) and the effect was particularly stronger for ER negative breast cancer (OR = 1.08, 95% CI = 1.01–1.16). There was no effect if we used pQTLs significant IVs (Table 2).

**Table 2:** Assessment of the association between HGF and breast cancer using Mendelian randomization analysis

HGF instrumental variables	Number of SNPs	Outcome	Beta	SE	OR (95% CI)	*P*-value
GWAS suggestive significant (1 × 10^−5^)	74	BC overall	0.046	0.021	1.05 (1.00, 1.09)	**0.0294**
	74	ER+ BC	0.045	0.025	1.05 (1.00, 1.10)	0.0733
	74	ER− BC	0.080	0.034	1.08 (1.01, 1.16)	**0.0200**
pQTL significant (1.7 × 10^−11^)	4	BC overall	−0.017	0.050	0.98 (0.89, 1.09)	0.7398
	4	ER+ BC	−0.048	0.067	0.95 (0.84, 1.09)	0.4790
	4	ER− BC	0.042	0.065	1.04 (0.92, 1.18)	0.5175

*BC* breast cancer. The instrumental variables were sourced from *metabolomips.org*. Statistically significant associations are in bold.

## Discussion

In this study, we prospectively evaluated the association between 92 plasma immuno-oncology biomarkers and breast cancer risk. The analysis revealed that ten proteins showed elevated levels prior to breast cancer diagnosis. These proteins include those involved in apoptosis (CASP8, FASLG), chemotaxis and inflammation (CCL4, CCL23, CXCL11, CCL3), cell proliferation and repair (LAP, TGF-β1, HGF), vascular remodeling (TEK), and immunosuppression (ARG1). Notably, the association of ARG1 with ER-negative breast cancer was stronger than that observed in the ER-positive group. Furthermore, although there were no differences in long-term and short-term risk effects, we noted elevated levels of CASP8, LAP, TGF-β1, HGF, and ARG1 in cases diagnosed within 5 years of follow-up compared to controls. Subsequent validation in the UKB-PPP successfully confirmed the association with elevated levels of HGF, particularly among postmenopausal women. Furthermore, MR analysis suggested a potential causal effect of HGF on breast cancer, particularly for the ER-negative subtype.

A higher level of circulating HGF was reported previously among breast cancer patients^18,19^, confirming HGF as a short-term risk biomarker for breast cancer. During the early stages of tumor progression, particularly in the atypical hyperplasia stage, HGF is significantly upregulated in the stromal compartment. This elevated expression of HGF activates the hepatocyte growth factor receptor (c-MET), which subsequently influences the mesenchymal-epithelial transition (MET) process in premalignant breast epithelial cells^20^. The HGF/c-MET signaling pathway then modulates the breast tumor microenvironment and drives malignant transformation by inducing c-MET dimerization and phosphorylation, which activates downstream pathways such as MAPK and PI3K^21^. These pathways are known to promote cancer cell proliferation, migration, and invasion, thereby enhancing tumor malignancy. Additionally, HGF induces the invasive properties of the ER-negative breast cancer cell by activating the Wnt-Met signaling pathway, confirming our findings in the stratified analysis and MR analysis for ER-negative breast cancer, and highlighting its critical role in the aggressive behavior of ER-negative breast cancer^22,23^. The significant detection of elevated HGF levels up to five years prior to diagnosis underscores the critical importance of early screening efforts, suggesting that timely interventions could be pivotal in improving patient outcomes.

Furthermore, our study found that HGF may mediate the effects of the HLI and BMI on breast cancer risk, which was more pronounced in postmenopausal women. An increase in BMI promotes inflammation in adipose tissue and elevates levels of HGF, which in turn enhances the expression of aromatase and the synthesis of estrogen^24^. Conversely, a higher HLI indicates a healthier lifestyle that correlates with a reduced risk of breast cancer. Notably, the marginally significant effect of physical activity suggests that regular exercise may further lower breast cancer risk by improving body composition, reducing inflammation, and lowering HGF levels. Therefore, promoting a healthy lifestyle that includes physical activity, alongside strategies to control BMI, reduce fat tissue inflammation, and regulate HGF signaling, may effectively lower breast cancer risk in the population.

Although the remaining ten proteins were not validated in the UKB-PPP, their biological functions suggest potential roles in breast cancer risk. In our study, LAP, TGF-β1, and HGF exhibited significant interactions, suggesting potential synergistic mechanisms in modulating breast cancer risk. TGF-β signaling pathways, which are known to promote angiogenesis and immune evasion^25^, have been shown in in vitro studies to trigger HGF-induced and MET-dependent migration^26^. These findings enhance our understanding of the potential collaborative roles of TGF-β1 and HGF in the initiation and progression of breast cancer. Notably, inhibitors of the HGF/c-Met and TGF-β signaling pathways, such as Cabozantinib^27^ and Fresolimumab^28^, have been involved in clinical trials for breast cancer and showed benefits in metastasis patients, suggesting their potential as novel therapies for breast cancer.

CASP8 showed the most significant statistical association with breast cancer risk, especially within 5 years before cancer diagnosis, consistent with findings from the KARMA study in Sweden^12^. CASP8 is a crucial initiator caspase in both the extrinsic and intrinsic apoptosis pathways. Its dysregulation can lead to evasion of apoptosis, tumor progression, and therapy resistance for cancers, highlighting its complex role in cancer biology^29^. FASLG also contributes to apoptosis by binding to its receptor FAS, which activates the extrinsic apoptotic pathway^30^. However, in tumors, FASLG can paradoxically promote immune evasion and tumor growth by inducing apoptosis in immune cells or through non-apoptotic signaling pathways, illustrating its dual role in cancer^31^.

ARG1 is an enzyme that depletes L-arginine, thereby suppressing T-cell function and promoting immune evasion, which facilitates tumor growth and progression^32^. TEK supports angiogenesis within tumors, thereby aiding growth and metastasis^33^. Additionally, TEK mediates interactions between endothelial and tumor cells, contributing to extracellular matrix remodeling and enhancing tumor cell migration^34^.

Proteins involved in chemotaxis and inflammation, including CCL3, CCL4, CXCL11, and CCL23, were only found to be associated with long-term breast cancer risk after five years, suggesting the long-term effect of inflammation on breast cancer risk. CCL3 and CCL4 are known to attract various immune cell types, including monocytes, macrophages, and T lymphocytes, which can influence both the innate and adaptive immune responses against tumors^35^. Moreover, CXCL11, a member of the CXC chemokine family, is particularly effective in recruiting T cells, thereby enhancing the cytotoxic immune response^36^. CCL23, which has both pro-inflammatory and immunomodulatory properties, can also affect the composition of the immune infiltrate within the tumor microenvironment, potentially promoting a more favorable environment for tumor progression^37^.

Our study demonstrates several notable strengths. The use of two nested case–control studies in prospective cohorts enhances the robustness of our findings and supports causal inferences regarding the relationship between HGF and breast cancer. Additionally, the application of MR analysis further solidifies the causal relationship between HGF and breast cancer. Nonetheless, our study is also subject to several limitations. First, some of the proteins discovered in TZL could not be validated in UKB-PPP. This discrepancy could be attributed to differences in ethnicity (Chinese vs. European), lifestyle behaviors and reproductive history (later age at menarche, earlier age at first birth, and lower rates of smoking and alcohol consumption in the TZL cohort), or sample status (fasting time before blood collection and storage time). The variations in lifestyle and reproductive history likely contribute to a higher proportion of ER-negative breast cancer in the TZL cohort (22.5%), which is consistent with findings from other regions in China^38–40^. In contrast, the UK population exhibits a lower proportion of ER-negative breast cancer (14.0%^41^). ER-positive breast cancer typically relies on hormone signaling pathways, while ER-negative breast cancer is more dependent on mechanisms such as cell proliferation, immune evasion, and metabolic reprogramming^42^. These differences in tumor characteristics, along with demographic variations, could potentially affect the generalizability of our findings when extrapolating from TZL to UKB-PPP. Second, given that circulating proteins were detected in plasma samples with EDTA, the use of EDTA may affect the stability and activity of certain proteins, potentially compromising detection accuracy and introducing biases. Future research should address these issues to enhance the reliability of our findings.

In summary, our research highlights the essential role of HGF as a biomarker for the early detection of breast cancer, particularly in postmenopausal women. Furthermore, HGF may also be connected to the biological mechanisms linking a healthy lifestyle and BMI to breast cancer risk. This insight paves the way for future studies to investigate targeted interventions that address the pathways influenced by promoting a healthy lifestyle and managing BMI, potentially resulting in more effective prevention strategies.

## Methods

### Study population

This study is based on two nested case–control studies in the Taizhou Longitudinal Study (TZL) cohort and the UK Biobank Pharma Proteomics Project (UKB-PPP). A total of 29,110 female adults enrolled in the TZL cohort between 2009 and 2014 were selected as the study population. Detailed information about the TZL cohort has been previously reported^43^. Follow-up continued until December 31, 2021, during which 252 breast cancer cases were identified using the linked medical insurance records. Twenty-four cases were excluded due to a prior history of breast cancer before enrollment. Breast cancer diagnosis was based on the International Classification of Diseases-10 code C50. Tumor characteristics for the patients were available after linkage to the hospital information system of Taizhou People’s Hospital. Each breast cancer case was matched with one cancer-free, living control based on age and time to breast cancer diagnosis. This study was approved by the Ethics Committee of Fudan University Taizhou Institute of Health Sciences (2021-B018) and Fujian Medical University (2022–2026). This study was performed in accordance with the Declaration of Helsinki, and informed consent was obtained from all participants.

### UKB-PPP project

UKB-PPP is a biopharmaceutical consortium characterizing the plasma proteomic profiles of 54,219 UK Biobank participants, among which the randomly selected sub-sample of 25,397 women who underwent Olink proteomics analysis was included with 1632 breast cancer cases. Participants with unknown ethnicity (*N* = 108) or a history of breast cancer (*N* = 751) were excluded, resulting in a dataset comprising 881 incident breast cancer cases. Each case was set to match a maximum of 10 cancer-free, living controls based on age, center, ethnicity, and time to breast cancer diagnosis. All the UKB participants gave informed consent, and the study was approved by the National Information Governance Board for Health and Social Care and the National Health Service North West Center for Research Ethics Committee (Ref: 11/NW/0382, 17 June 2011).

### Proteomics assay

For TZL, baseline plasma samples were collected and stored at −80 °C for subsequent analysis, using the Olink Target Immuno-oncology panel, which encompasses 92 unique protein biomarkers. Prior to analysis, the samples were thawed in a controlled gradient manner and randomized onto a 96-well plate, with each well containing 35 μL of plasma. These prepared samples were then transported on dry ice to the Olink laboratory located in Shanghai, China. Quantification of protein levels was achieved through the proximity extension assay (PEA) technique, generating normalized protein expression (NPX) values for each protein. To maintain assay integrity and ensure sample quality, four internal controls were incorporated into each sample. The quality control (QC) protocol involved assessing the standard deviation of these internal controls across each sample plate. Only plates with a standard deviation below 0.2 NPX were deemed acceptable for data reporting. Furthermore, individual sample quality was evaluated by comparing their deviation from the median value of the controls. Samples were considered to pass QC if their deviation was less than 0.3 NPX from the median. After QC, 33 samples (20 cases, 13 controls) were excluded. NPX values below the limit of detection (LOD) were retained. The final dataset included 195 matched pairs for analysis.

In UKB-PPP, 2923 proteins were analyzed using the same Olink protocol, which includes the 92 Immuno-oncology protein biomarkers that we have explored. Comprehensive details regarding the proteomic profiles of the UKB-PPP have been reported^44^.

### Statistical analysis

Baseline characteristics, including income, alcohol consumption, smoking status, BMI, waist circumference, height, age at menarche, menopausal status, number of births, and family history of breast cancer, were compared between breast cancer cases and their matched controls using conditional logistic regression. To address missing data, multiple imputation was performed using chained equations with 10 iterations each^45^. Sensitivity analyses showed similar results with and without imputation. Thus, the results from the multiple imputed datasets were reported. Subsequently, the protein expression values were normalized via rank-based inverse normal transformation. Conditional logistic regression analysis was employed to assess the association between each protein and breast cancer incidence, with adjustments made for potential confounders including income, age at menarche, menopausal status, number of births, BMI and height. Benjamini–Hochberg correction was applied to adjust *P*-values for the association between 92 proteins and breast cancer risk, with a threshold of false discovery rate (FDR) <0.05. Findings confirmed by both the original and imputed data were considered for further analysis.

To explore nonlinear relationships between differentially expressed proteins and breast cancer, we employed restricted cubic spline (RCS) models with four knots, using the average protein level as a reference. Furthermore, we incorporated stratified analyses based on tumor characteristics (including ER status, the TNM stage, tumor size, and lymph node (LN) metastasis), menopausal status and the time from baseline to breast cancer diagnosis to examine variations in these associations. Box plots illustrated the comparison of protein level distributions at baseline, categorized by the number of years prior to breast cancer diagnosis and further stratified by menopausal status.

Furthermore, we included multiple proteins in the same regression model simultaneously, grouped by their pathway-specific functions. If two or more proteins within a group were associated with breast cancer, their interaction terms were subsequently incorporated into the model to assess potential combined effects. Additionally, we utilized the Search Tool for the Retrieval of Interacting Genes/Proteins (STRING, https://string-db.org/) to analyze the interaction relationships among the identified proteins^46^. Subsequently, these proteins were subjected to Gene Ontology (GO) and Kyoto Encyclopedia of Genes and Genomes (KEGG) pathway analyses using the Bioconductor package, clusterProfiler 4.0, in R software^47^.

In UKB-PPP, conditional logistic regression was employed to examine the associations between identified proteins and breast cancer, with adjustments for Townsend Deprivation Index (TDI), alcohol consumption, smoking status, age at menarche, menopausal status, number of births, BMI, height, hormone replacement therapy (HRT) history, family history of breast cancer, and fasting time at blood draw. We excluded cases that lacked protein detection values (*N* = 25, on average) or had no matched controls (*N* = 2). NPX values of each protein were standardized using rank-based inverse normal transformation. For validated proteins, their roles as mediators for key modifiable risk factors for breast cancer (including the healthy lifestyle index (HLI), consisting mainly of lifestyle factors, BMI, physical activity assessed in metabolic equivalent of task (MET), and history of HRT) were examined using the Stata module med4way^48^.

### Mendelian randomization analysis

Concerning the proteins validated in UKB-PPP, a two-sample MR analysis was conducted to assess the causal effects of the identified protein biomarkers on breast cancer risk. This analysis utilized pQTLs derived from genome-wide association studies (GWAS) of the UKB. Independent single-nucleotide polymorphisms (SNPs) with pQTL GWAS significance (*P* < 1.7 × 10^−11^) and GWAS suggestive significance (*P* < 10^−5^) were selected as instrumental variables (IVs). The clumping method was further used to exclude the IV in linkage disequilibrium (LD) with *r*^2^ < 0.01. GWAS summary statistics for overall breast cancer and by ER status were downloaded from the Breast Cancer Association Consortium (BCAC), encompassing 122,977 cases and 105,974 controls of European ancestry^49^. The inverse variance weighted (IVW) method was applied to estimate the causal effect of proteins on breast cancer.

All statistical analyses and visualizations were performed using R (version 4.2.0) and Stata (version 15.1).

## Supplementary information

Supplementary Material

## Data Availability

Summary statistics for HGF pQTLs are available at *metabolomips.org*. Summary statistics for GWAS of intrinsic-like subtypes of breast cancer were downloaded from the BCAC website (http://bcac.ccge.medschl.cam.ac.uk/bcacdata/). Codes generated or used during the study are available from the corresponding author by request.
