# Supplementary material for: Association of circulating immuno-oncology biomarkers with breast cancer risk: insights from two prospective cohorts
Source: NPJ Precis Oncol. 2025 Jul 15;9:238. doi: 10.1038/s41698-025-01019-z (PMC12263894; doi:10.1038/s41698-025-01019-z)
Supplement: Supplementary file 1 — Supplementary Material [file 41698_2025_1019_MOESM1_ESM.pdf]

Supplementary materials

Table S1. Adjusted association between 11 proteins and breast cancer in original data and multiply imputed data analysis in the TZL cohort.

Protein	Original data (N = 390)			Multiply imputed data (N = 390)		
	OR (95% CI)	*P* [a]	FDR	OR (95% CI)	*P* [b]	FDR
CASP8	3.71 (2.50, 5.50)	7.67E-11	**7.06E-09**	3.71 (2.49, 5.54)	1.39E-10	**1.28E-08**
CCL4	1.73 (1.31, 2.28)	0.0001	**0.0057**	1.72 (1.30, 2.27)	0.0001	**0.0056**
LAP TGF-β1	1.44 (1.15, 1.81)	0.0016	**0.0238**	1.49 (1.18, 1.89)	0.0009	**0.0167**
HGF	1.52 (1.18, 1.96)	0.0012	**0.0224**	1.54 (1.19, 1.98)	0.0009	**0.0167**
CCL23	1.51 (1.19, 1.93)	0.0008	**0.0195**	1.51 (1.19, 1.93)	0.0008	**0.0167**
MMP7	0.72 (0.57, 0.90)	0.0046	**0.0468**	0.69 (0.55, 0.87)	0.0018	**0.0254**
FASLG	1.44 (1.14, 1.82)	0.0020	**0.0266**	1.44 (1.14, 1.82)	0.0022	**0.0254**
TEK	1.53 (1.20, 1.96)	0.0007	**0.0195**	1.46 (1.15, 1.87)	0.0023	**0.0254**
ARG1	1.44 (1.13, 1.84)	0.0029	**0.0330**	1.46 (1.14, 1.87)	0.0025	**0.0254**
CXCL11	1.35 (1.09, 1.67)	0.0062	**0.0480**	1.38 (1.11, 1.72)	0.0036	**0.0327**
CCL3	1.41 (1.10, 1.80)	0.0063	**0.0480**	1.41 (1.10, 1.80)	0.0064	**0.0488**

[a] Adjusted for income, age at menarche, menopausal status and BMI. [b] Adjusted for income, age at menarche, menopausal status, number of births , BMI and height.

Table S2. Pathway-specific functional analysis of multiple proteins in association with incident breast cancer risk.

Functional classification	OR (95% CI)	*P*-value [a]
Apoptosis		
CASP8	3.63 (2.41, 5.46)	**< 0.001**
FASLG	1.08 (0.81, 1.45)	0.601
Growth factor		
LAP TGF-β1	1.31 (1.01, 1.71)	**0.044**
HGF	1.34 (1.00, 1.78)	**0.049**
Chemokine		
CCL4	1.75 (1.14, 2.69)	**0.010**
CCL23	1.36 (1.04, 1.77)	**0.024**
CXCL11	1.15 (0.89, 1.48)	0.283
CCL3	0.79 (0.53, 1.19)	0.264
Angiogenesis		
TEK	1.38 (1.08, 1.77)	**0.011**
ARG1	1.38 (1.07, 1.77)	**0.012**

[a] Adjusted for income, age at menarche, menopausal status, number of births, BMI and height.

Table S3. Pathway-specific functional analysis of multiple proteins (including protein-protein interactions) in association with incident breast cancer risk.

Functional classification	OR (95% CI)	*P*-value [a]
Growth factor		
LAP TGF-β1	1.46 (1.09, 1.97)	**0.012**
HGF	1.37 (1.02, 1.85)	**0.035**
LAP TGF-β1*HGF	1.43 (1.14, 1.81)	**0.002**
Chemokine		
CCL4	1.84 (1.19, 2.84)	**0.006**
CCL23	1.38 (1.05, 1.81)	**0.019**
CXCL11	1.15 (0.89, 1.48)	0.278
CCL3	0.78 (0.52, 1.18)	0.240
CCL4*CCL23	1.28 (0.99, 1.66)	0.060
Angiogenesis		
TEK	1.37 (1.06, 1.76)	**0.014**
ARG1	1.4 (1.09, 1.8)	**0.009**
TEK*ARG1	1.12 (0.86, 1.46)	0.403

[a] Adjusted for income, age at menarche, menopausal status, number of births, BMI and height.

Table S4. Mediation analysis of HGF in the relationship between the Health Lifestyle Index and its components in relation to breast cancer among postmenopausal females in UKB-PPP.

Exposure variable	β (total)	*P* (total)	β (CDE)	*P* (CDE)	HGF mediation proportion	*P* (mediation)
HLI	-0.207	<0.001	-0.161	<0.001	27.17%	**0.017** [a]
BMI	0.048	<0.001	0.038	0.001	19.79%	**0.037** [b]
Physical activity MET	-0.103	0.011	-0.093	0.023	20.38%	0.076 [b]
Alcohol consumption	0.840	0.019	1.061	0.005	-6.44%	0.107 [c]
Smoking status	0.138	0.469	0.062	0.755	52.18%	0.530 [d]
Diet quality score	-0.054	0.648	-0.058	0.625	13.62%	0.678 [e]

As presented in the main text, the analysis revealed a statistically significant mediation effect of HGF between HLI and BMI, as well as a marginally significant mediation effect concerning physical activity measured in MET, in relation to breast cancer. [a] Adjusted for TDI, age at menarche, number of births, height, HRT history, family history of breast cancer, and fasting time at blood draw. [b] Adjusted for TDI, alcohol consumption, smoking status, age at menarche, number of births, height, HRT history, family history of breast cancer, and fasting time at blood draw. [c] Adjusted for TDI, smoking status, age at menarche, number of births, BMI, height, HRT history, family history of breast cancer, and fasting time at blood draw. [d] Adjusted for TDI, alcohol consumption, age at menarche, number of births, BMI, height, HRT history, family history of breast cancer, and fasting time at blood draw. [e] Adjusted for TDI, alcohol consumption, smoking status, age at menarche, number of births, BMI, height, HRT history, family history of breast cancer, and fasting time at blood draw. HLI: Healthy Lifestyle Index. CDE: controlled direct effect. MET: metabolic equivalent of task. TDI: Townsend Deprivation Index. HRT: hormone replacement therapy.

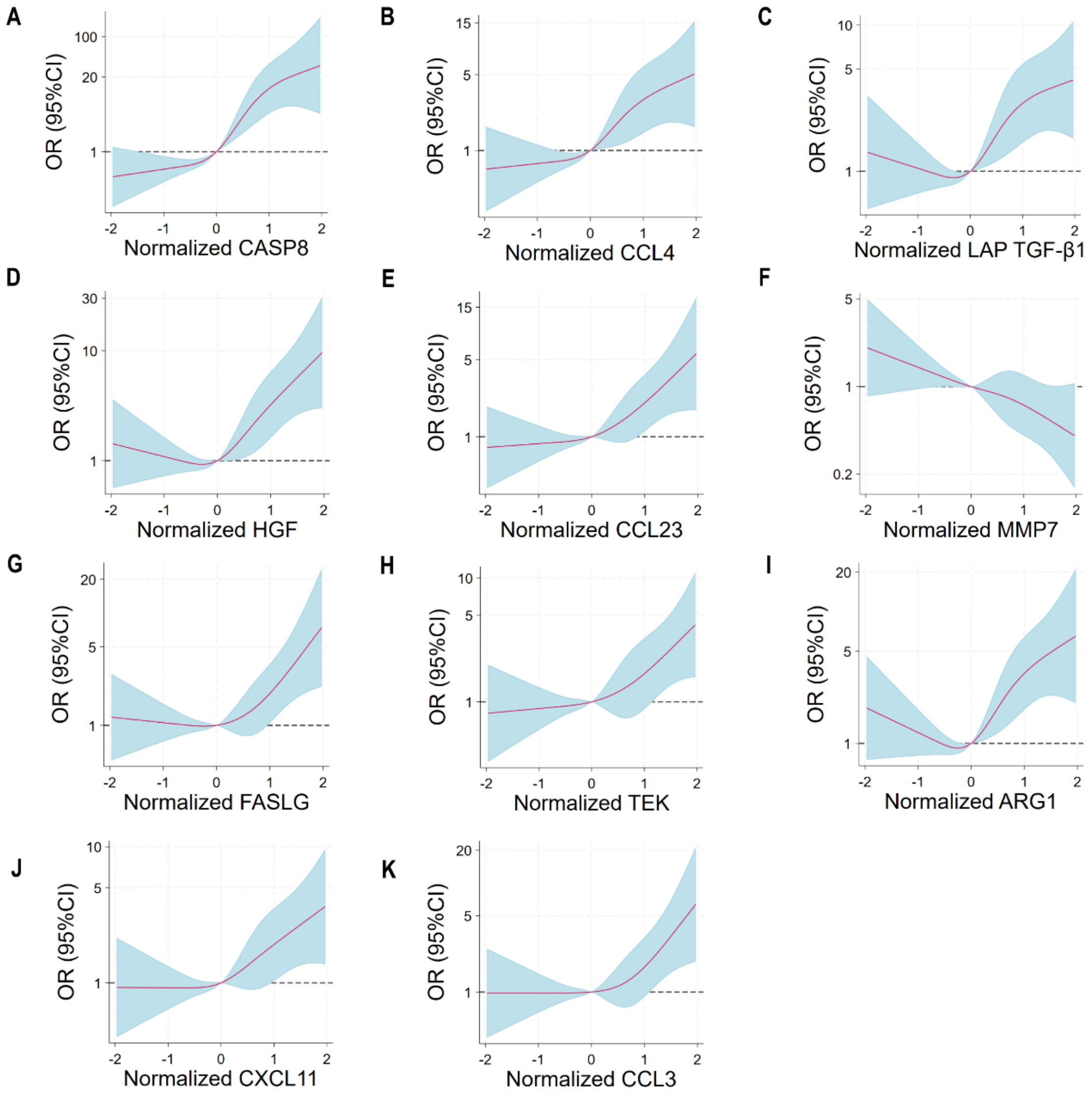

Figure S1. The Restricted Cubic Spline plot of 11 proteins significantly associated with incident breast cancer in the TZL cohort. Adjusted for income, age at menarche, menopausal status, number of births, BMI and height. The proteins analyzed include: CASP8 (A), CCL4 (B), LAP TGF-β1 (C), HGF (D), CCL23 (E), MMP7 (F), FASLG (G), TEK (H), ARG1 (I), CXCL11 (J), and CCL3 (K).

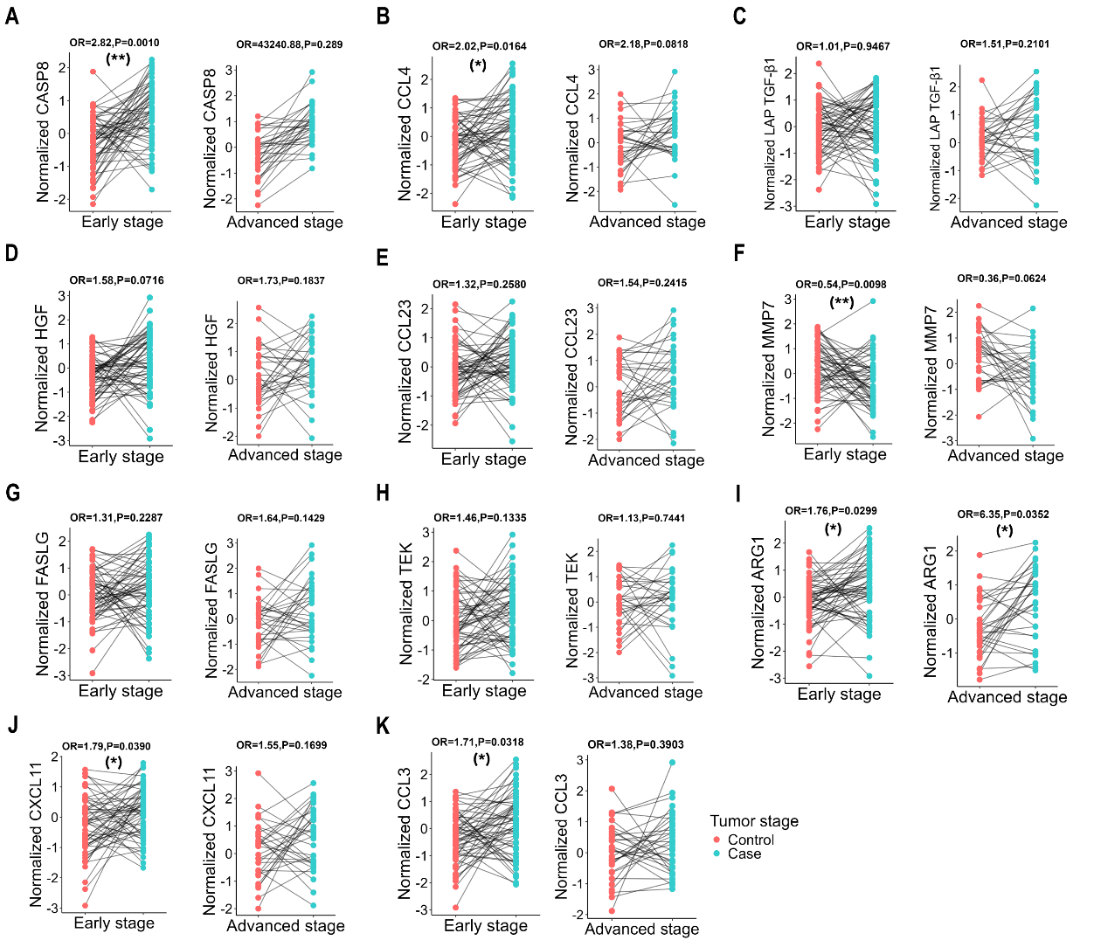

Figure S2. Associations between 11 proteins and incident breast cancer stratified by tumor TNM stage in the TZL cohort study (early stage: 57 cases; advanced stage: 32 cases). Early stage was defined as TNM stages 0-IIA, while advanced stage included stages IIB-III. Adjusted for age at menarche, menopausal status, number of births, BMI and height. The proteins analyzed include: CASP8 (A), CCL4 (B), LAP TGF-β1 (C), HGF (D), CCL23 (E), MMP7 (F), FASLG (G), TEK (H), ARG1 (I), CXCL11 (J), and CCL3 (K). Statistical significance is denoted as follows: *$P < 0.05$, **$P < 0.01$, and ***$P < 0.001$.

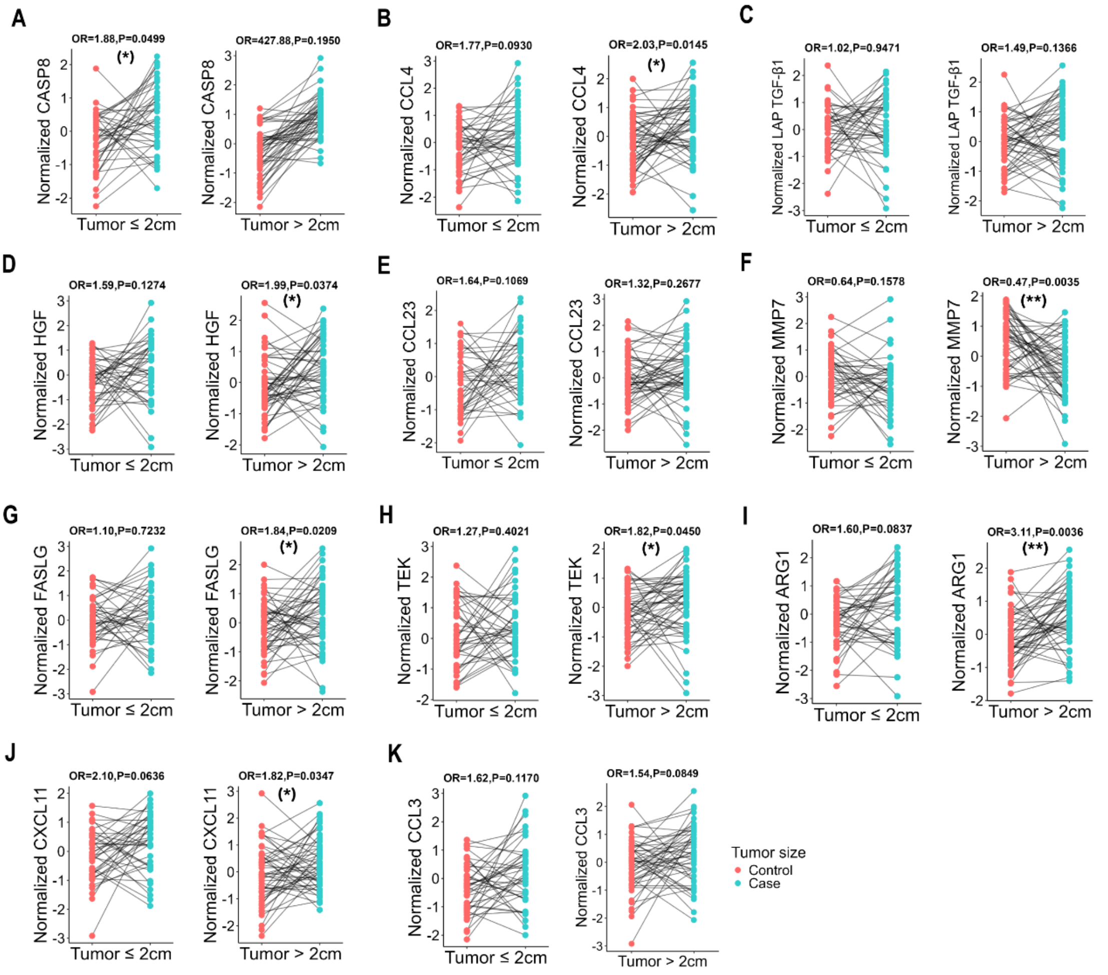

Figure S3. Associations between 11 proteins and incident breast cancer stratified by tumor size in the TZL cohort study (tumor size ≤ 2cm: 40 cases; tumor size > 2cm: 49 cases). Adjusted for age at menarche, menopausal status, number of births, BMI and height. The proteins analyzed include: CASP8 (A), CCL4 (B), LAP TGF-β1 (C), HGF (D), CCL23 (E), MMP7 (F), FASLG (G), TEK (H), ARG1 (I), CXCL11 (J), and CCL3 (K). Statistical significance is denoted as follows: *$P < 0.05$, **$P < 0.01$, and ***$P < 0.001$.

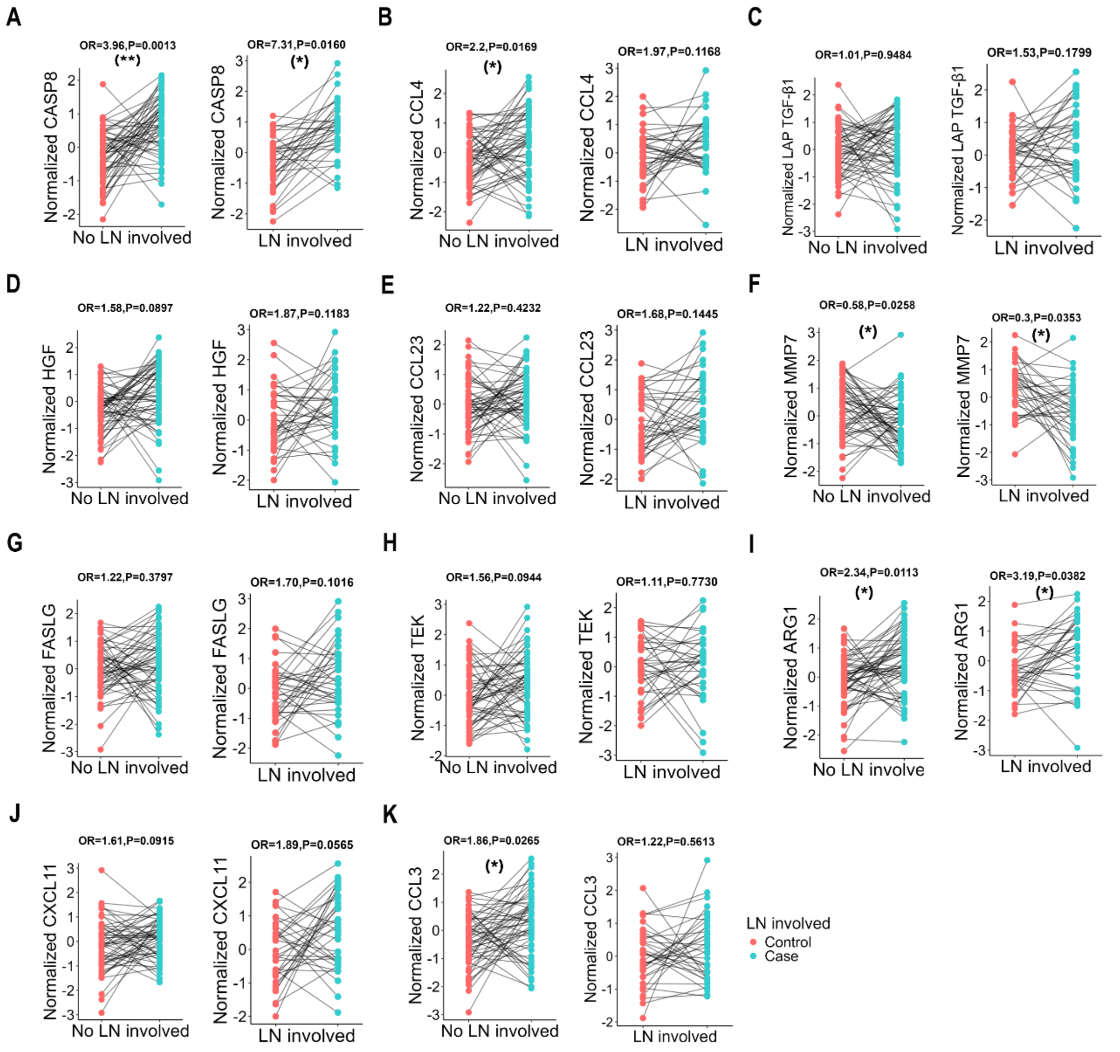

Figure S4. Associations between 11 proteins and incident breast cancer stratified by lymph node (LN) metastasis in the TZL cohort study (no LN metastasis: 54 cases; LN metastasis: 35 cases). Adjusted for age at menarche, menopausal status, number of births, BMI and height. The proteins analyzed include: CASP8 (A), CCL4 (B), LAP TGF-β1 (C), HGF (D), CCL23 (E), MMP7 (F), FASLG (G), TEK (H), ARG1 (I), CXCL11 (J), and CCL3 (K). Statistical significance is denoted as follows: *$P < 0.05$, **$P < 0.01$, and ***$P < 0.001$.

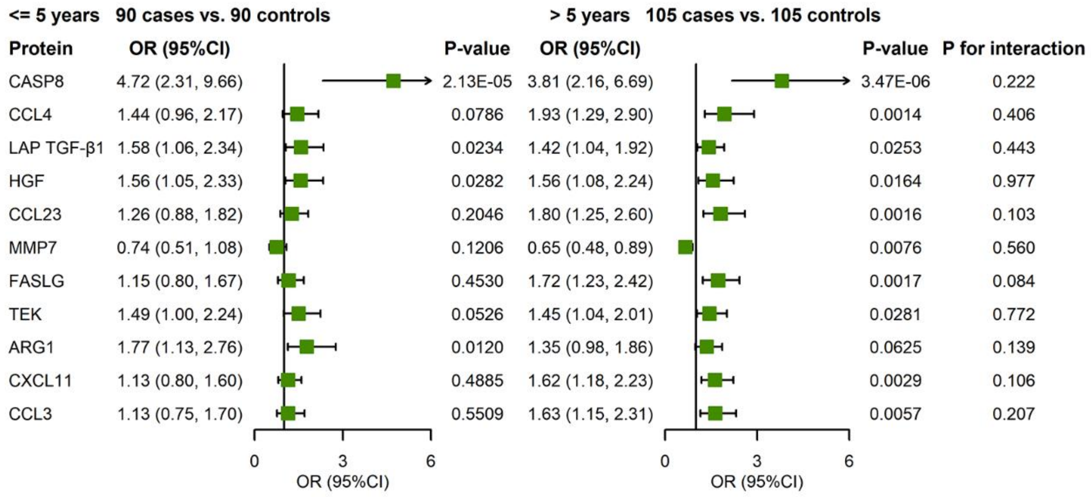

Figure S5. Comparison of 11 proteins between controls and incident breast cancer cases stratified by time-to-diagnosis from baseline in the TZL cohort. Adjusted for income, age at menarche, menopausal status, number of births, BMI and height.

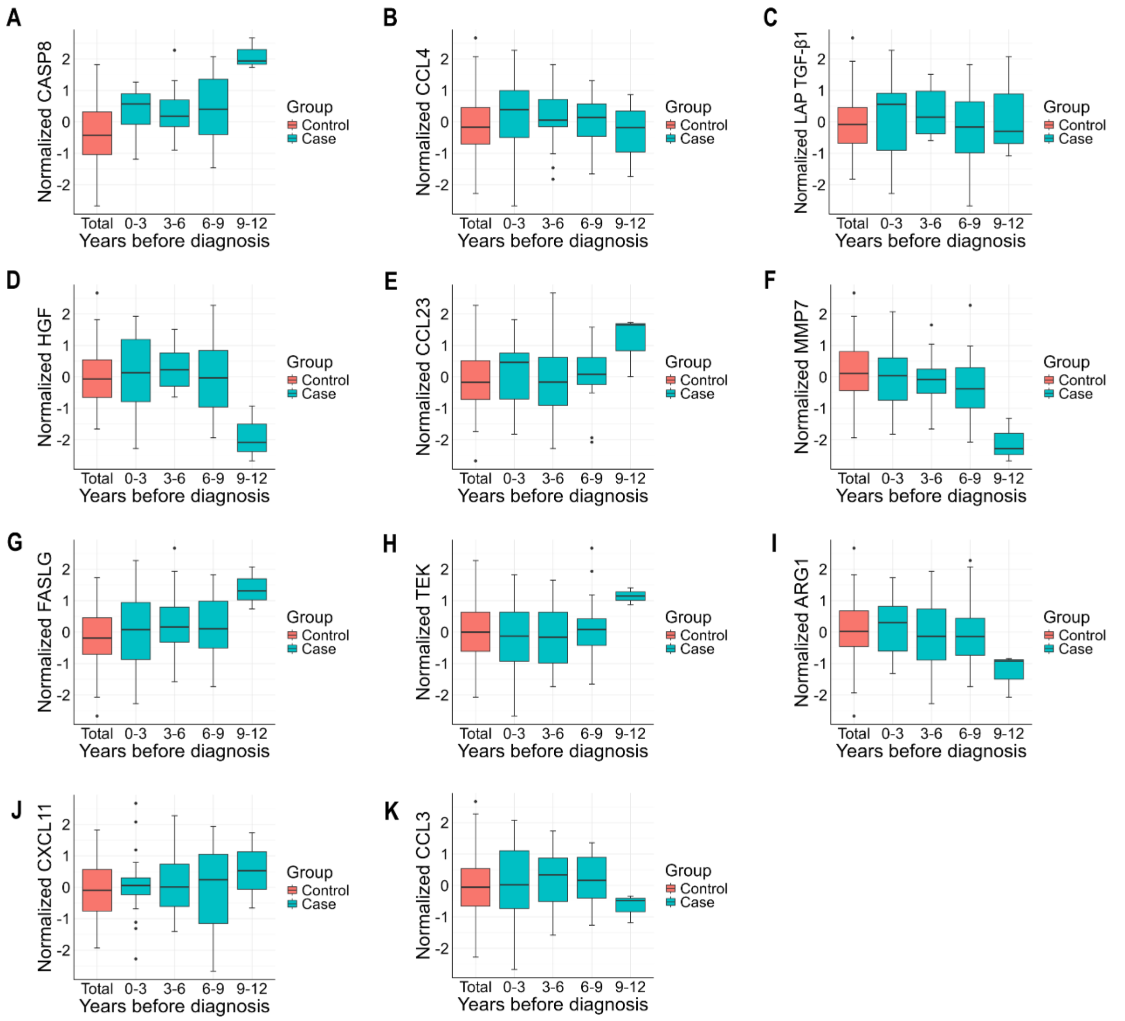

Figure S6. The box plots illustrate the distributions of the 11 identified proteins based on the years before breast cancer diagnosis among premenopausal women in the TZL cohort, organized by three-year intervals. The proteins analyzed include: CASP8 (A), CCL4 (B), LAP TGF-β1 (C), HGF (D), CCL23 (E), MMP7 (F), FASLG (G), TEK (H), ARG1 (I), CXCL11 (J), and CCL3 (K).

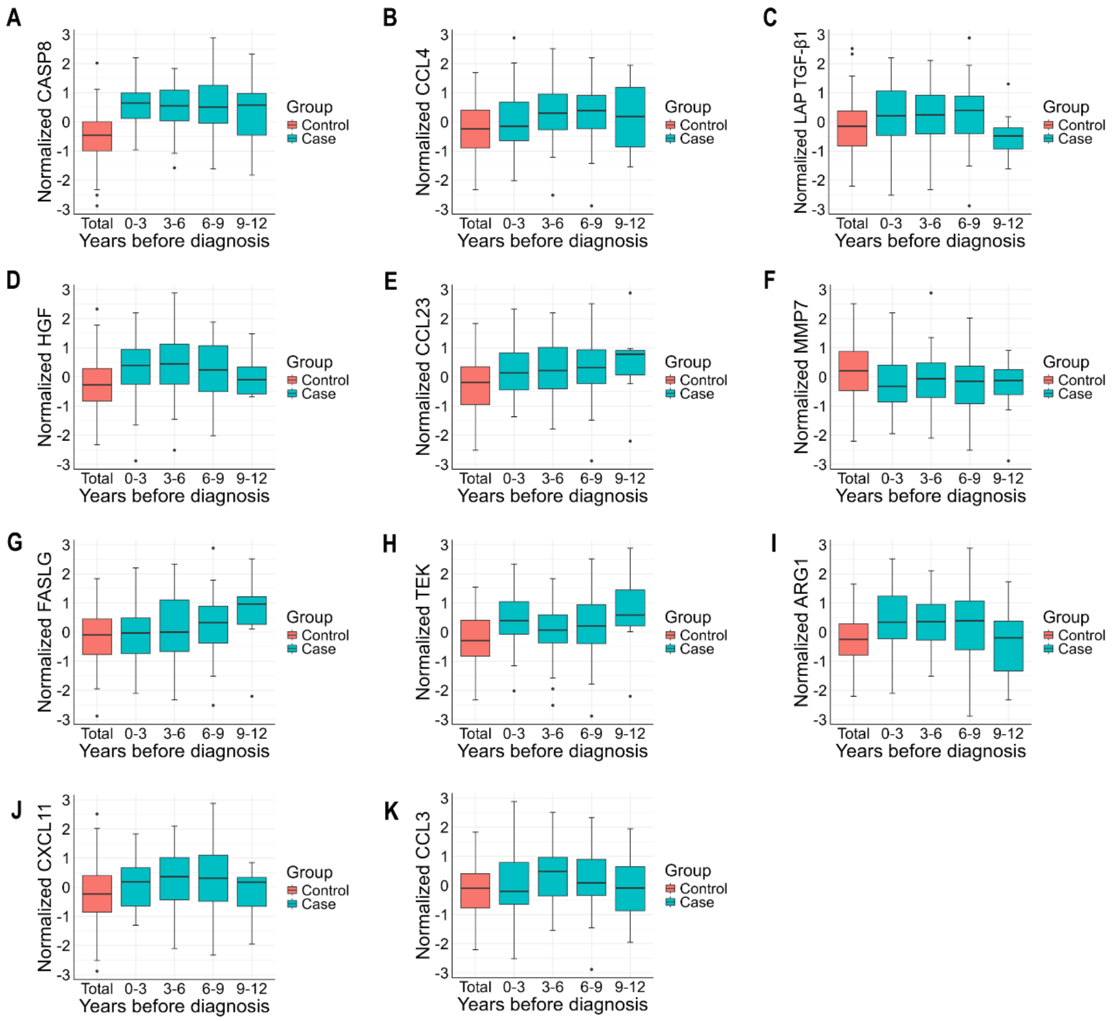

Figure S7. The box plots illustrate the distributions of the 11 identified proteins based on the years before breast cancer diagnosis among postmenopausal women in the TZL cohort, organized by three-year intervals. The proteins analyzed include: CASP8 (A), CCL4 (B), LAP TGF-β1 (C), HGF (D), CCL23 (E), MMP7 (F), FASLG (G), TEK (H), ARG1 (I), CXCL11 (J), and CCL3 (K).

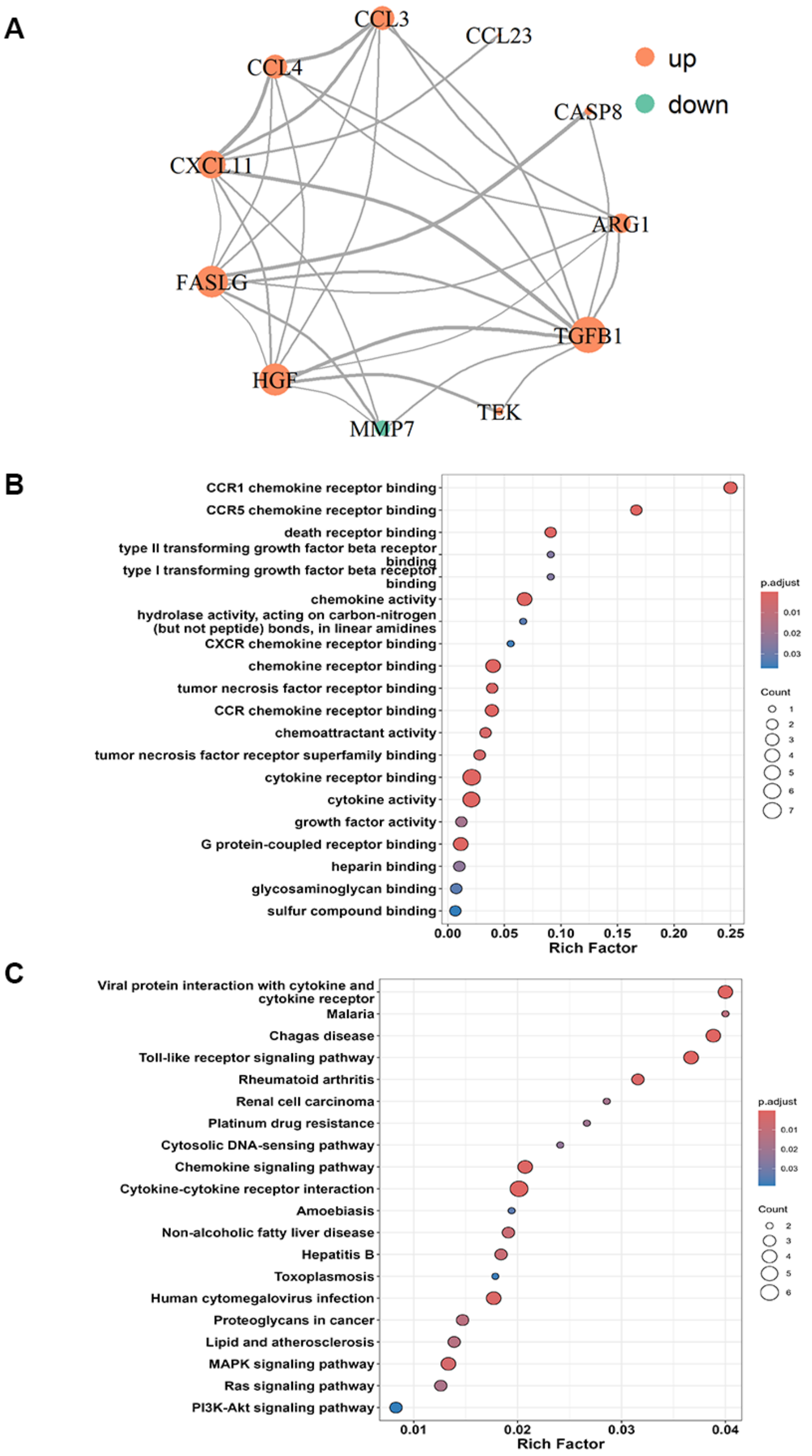

Figure S8. Bioinformatics analysis of 11 proteins significantly associated with incident breast cancer. (A) Protein-Protein Interaction analysis, (B) Gene Ontology analysis, (C) Kyoto Encyclopedia of Genes and Genomes pathway analysis.

Time-to-diagnosis	N (case)	N (control)	OR (95%CI)	P-value
**All females**				
**<= 5 years**				
CASP8	359	2946	0.89 (0.79, 1.00)	0.0515
CCL4	360	2979	1.05 (0.93, 1.18)	0.4616
LAP TGF-β1	360	2979	1.00 (0.89, 1.12)	0.9869
HGF	360	2963	1.09 (0.96, 1.24)	0.1710
CCL23	354	2933	0.96 (0.85, 1.09)	0.5361
MMP7	361	2961	1.05 (0.93, 1.18)	0.4452
FASLG	360	2963	0.88 (0.78, 0.99)	0.0297
TEK	355	2945	0.95 (0.85, 1.07)	0.4085
ARG1	363	3024	0.92 (0.82, 1.03)	0.1656
CXCL11	360	2975	0.94 (0.83, 1.05)	0.2740
CCL3	354	2918	0.97 (0.86, 1.11)	0.6871
**> 5 years**				
CASP8	501	4200	1.03 (0.93, 1.13)	0.6142
CCL4	494	4094	0.93 (0.84, 1.03)	0.1705
LAP TGF-β1	497	4158	0.97 (0.88, 1.07)	0.5667
HGF	498	4199	1.04 (0.94, 1.16)	0.4370
CCL23	477	3964	1.01 (0.91, 1.12)	0.8401
MMP7	498	4153	1.00 (0.90, 1.11)	0.9600
FASLG	498	4199	0.95 (0.86, 1.06)	0.3712
TEK	495	4137	0.99 (0.89, 1.09)	0.7831
ARG1	503	4188	0.99 (0.89, 1.09)	0.7862
CXCL11	499	4185	1.00 (0.91, 1.11)	0.9591
CCL3	492	4087	0.96 (0.87, 1.07)	0.5082
**Premenopausal females**				
**<= 5 years**				
CASP8	75	428	1.01 (0.78, 1.32)	0.9257
CCL4	75	411	1.06 (0.81, 1.37)	0.6883
LAP TGF-β1	76	433	0.88 (0.68, 1.14)	0.3426
HGF	76	435	0.87 (0.66, 1.15)	0.3310
CCL23	76	437	1.08 (0.82, 1.42)	0.5960
MMP7	75	438	1.01 (0.75, 1.34)	0.9657
FASLG	76	435	0.79 (0.59, 1.05)	0.1071
TEK	75	435	0.98 (0.76, 1.26)	0.8588
ARG1	76	419	0.84 (0.65, 1.08)	0.1668
CXCL11	74	422	0.84 (0.64, 1.10)	0.1995
CCL3	74	411	0.79 (0.59, 1.06)	0.1211
**> 5 years**				
CASP8	111	654	0.98 (0.80, 1.20)	0.8567
CCL4	108	640	0.94 (0.76, 1.15)	0.5296
LAP TGF-β1	107	629	0.98 (0.78, 1.22)	0.8418
HGF	107	642	0.92 (0.74, 1.14)	0.4439
CCL23	107	665	0.99 (0.79, 1.24)	0.9249
MMP7	109	661	0.96 (0.75, 1.23)	0.7387
FASLG	107	642	0.85 (0.67, 1.08)	0.1825
TEK	111	663	1.06 (0.86, 1.32)	0.5669
ARG1	112	665	0.82 (0.66, 1.02)	0.0746
CXCL11	110	655	0.94 (0.77, 1.16)	0.5888
CCL3	107	662	0.98 (0.78, 1.22)	0.8242
**Postmenopausal females**				
**<= 5 years**				
CASP8	266	1957	0.86 (0.75, 0.99)	0.0320
CCL4	267	2009	1.04 (0.90, 1.19)	0.6113
LAP TGF-β1	266	1990	1.04 (0.91, 1.19)	0.5231
HGF	266	1994	1.19 (1.03, 1.37)	0.0183
CCL23	261	1942	0.96 (0.83, 1.10)	0.5390
MMP7	268	1991	1.05 (0.92, 1.20)	0.4847
FASLG	266	1994	0.88 (0.77, 1.01)	0.0694
TEK	262	1949	0.95 (0.83, 1.09)	0.4776
ARG1	269	2033	0.94 (0.82, 1.07)	0.3403
CXCL11	268	2002	0.92 (0.80, 1.05)	0.2215
CCL3	262	1949	1.00 (0.86, 1.16)	0.9834
**> 5 years**				
CASP8	370	2921	1.05 (0.94, 1.18)	0.3726
CCL4	366	2837	0.94 (0.84, 1.06)	0.3416
LAP TGF-β1	370	2901	0.99 (0.88, 1.11)	0.8169
HGF	371	2942	1.07 (0.95, 1.21)	0.2801
CCL23	352	2716	1.02 (0.90, 1.14)	0.7779
MMP7	369	2872	0.97 (0.86, 1.10)	0.6565
FASLG	371	2942	1.02 (0.91, 1.15)	0.7050
TEK	364	2862	0.99 (0.89, 1.12)	0.9173
ARG1	372	2912	1.03 (0.92, 1.16)	0.6178
CXCL11	369	2911	1.01 (0.90, 1.14)	0.8430
CCL3	365	2835	0.99 (0.87, 1.12)	0.8204

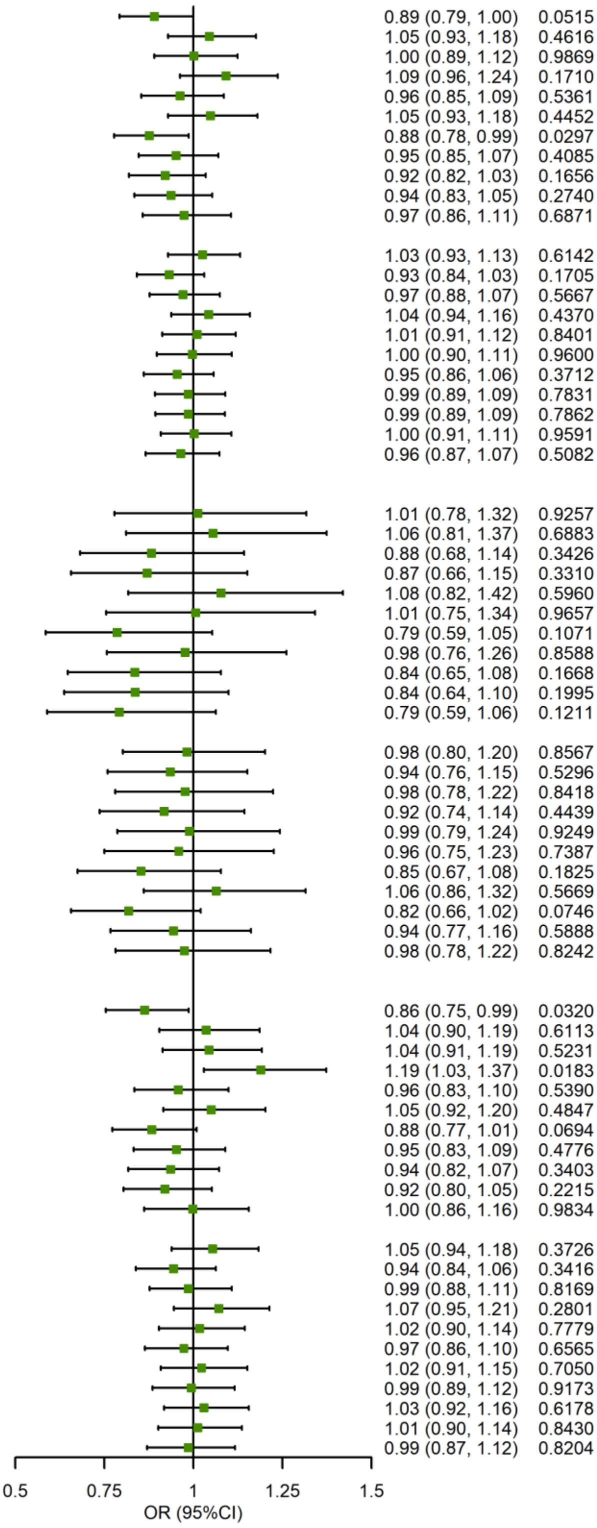

Figure S9. Comparison of 11 proteins between controls and incident breast cancer cases stratified by time-to-diagnosis from baseline in UKB-PPP. Adjusted for TDI, alcohol consumption, smoking status, age at menarche, menopausal status (for all females), number of births, BMI, height, HRT history, family history of breast cancer, and fasting time at blood draw.
